# Animus: human-embodied animals

**DOI:** 10.1136/jme-2022-108817

**Published:** 2023-10-06

**Authors:** Julian Savulescu, Tsutomu Sawai

**Affiliations:** 1 Centre for Biomedical Ethics, Yong Loo Lin School of Medicine, National University of Singapore, Singapore; 2 Biomedical Research Group, Murdoch Children's Research Institute, Parkville, Victoria, Australia; 3 Faculty of Philosophy, University of Oxford, Oxford, UK; 4 Graduate School of Humanities and Social Sciences, Hiroshima University, Higashihiroshima, Hiroshima, Japan; 5 Institute for the Advanced Study of Human Biology (ASHBi), Kyoto University, Kyoto, Japan

**Keywords:** ethics- research, animals, chimera

## Abstract

We review recent research to introduce human brain organoids into the brains of infant rats. This research shows these organoids integrate and function to affect behaviour in rats. We argue that this raises issues of moral status that will imminently arise and must be addressed through functional studies of these new life forms. We situate this research in the broader context of the biological revolution, arguing we already have the technological power to create fully human embodied animals. This raises profound, so far unaddressed ethical issues which call for urgent attention.

 In the 1958 horror film, *The Fly*, a scientist is experimenting with an atomic transporter. A fly enters his laboratory and his atoms get mixed up with the fly, turning him into a monstrous creature part fly, part human.

Human-animal chimaeras, characterised by the incorporation of human components into animals, have long been the subject of scientific and ethical debate. In terms of historical precedent, the advent of xenotransplantation was documented in 1906, marking a landmark episode of kidney transplants from sheep and pigs to human recipients. Remarkably, there were instances of sheep blood transfusions into humans more than two centuries before this event.^1^

In the present era, fears of human-animal chimaeras, as depicted in *The Fly*, have moved from the realm of speculative science fiction to compelling reality.

## Human brain organoids in rats

Brain organoids are independent nerve (neuronal) structures, which can be made from a person’s cells, such as their skin or blood cells, using induced pluripotent stem (iPS) cell technology to respecialise the cells. Although they cannot yet replicate a full brain, they resemble features or parts of a human brain. Since the pioneering transplantation of human brain organoids into animals in 2017,^2^ numerous transplantation studies have been conducted.^3^ Recently, Sergiu Pașca, a leading scientist in the field of brain organoid research, along with his research group, has achieved a noteworthy milestone by successfully transplanting human brain organoids into rats.^4^ They have shown that these human brain organoids integrate into the rat brain and function, even being capable of affecting the behaviour of rats. Up to one-sixth of the rat cortex was human.^4^ In terms of their biology, these are ‘humanised rats’. (However, this terminology has invited critique for its inherent ambiguity and potential for misconstrual, giving rise to undue anthropomorphic interpretations. Therefore, a more precise and contextually fitting descriptor for rats implanted with human brain organoids may be ‘enhanced’ rats, a term that effectively eliminates any misleading anthropomorphic connotations.)

This is an exciting discovery for science. It will allow brain organoids to grow bigger than they have in a lab, and opens up many possibilities of understanding how early human neurons develop and form the brain, and what goes wrong in disease. Indeed, the rat models showed the neuronal defects related to one severe disease, Timothy Syndrome. This is one step further along the long road to making progress in brain disease, which has proved so intransigent so far.

The goals of this research are laudable. But at the same time, it calls for new standards to be set for future research. It opens the door to more elaborate or ambitious research which would raise significant ethical issues.

## Uncertainties of human brain organoid transplantation

National and international authorities have thus far maintained a sceptical stance concerning the potential for immature brain organoids to attain consciousness or achieve cognitive enhancement through implantation into animal brains in the foreseeable future.^5 6^ Yet, evaluations regarding the consciousness of brain organoids are fundamentally linked to perspectives on consciousness.^7 8^

From the perspective of integrated information theory, the inherent capacity of a system to amalgamate information is deemed a sufficient condition for consciousness.^9^ This hypothesis posits that even current immature brain organoids, if they display complex neural activity patterns, may meet the criteria for consciousness attribution. Conversely, Global Workspace Theory proposes that the emergence of consciousness within a brain is dependent on the presence of functional connectivity between cortical regions, facilitated by long-range neuronal projections.^10^ Presently, existing immature brain organoids do not satisfy this criterion, and it would not be easy to do so in the future.

Regardless of whether they are conscious or not, embedding human brain organoids with complex neural activity patterns into a conscious brain’s structure might proportionally correlate with an enhancement in cognitive and mental capabilities. In the Pașca study, the brain organoid was introduced into the sensory part of the animal brain. As more sophisticated brain organoids are introduced in the future, or if such brain organoids are introduced affecting more key areas of the brain, the rat brain may acquire more advanced consciousness, including higher rational capacities or self-consciousness. This would raise issues of how such ‘enhanced’ rats ought to be treated. They might begin to acquire rights and interests closer to primates than rodents. It would be important to not treat them as rats, just because they look like rats, if their brains are significantly enhanced. Cognitive enhancement in chimeric animals has been a controversial issue in human-animal chimaera research since 2003, with many arguing for the moral consideration of animals with enhanced abilities as a result of scientific research.^11 12^ It raises issues of moral status.^13^

This requires discussion and boundaries set around what kinds of organoids can be implanted and what key sites would be targets for enhancement of capacities that matter to moral status. It also requires us to decide what confers what kind of moral status.^13^ This is a combined scientific and ethical challenge. The ethical implications of potentially imbuing these entities with distinctively human capacities, such as the ability to make moral judgments or bear responsibility for actions, are still unclear. Renowned ethicist Insoo Hyun bifurcates this concept into two categories: ‘moral humanisation’, in which an animal brain, despite containing human neurons, functions similarly to a human; and ‘biological humanisation’, where despite the presence of human neurons, there is no functional resemblance. This dichotomy underlines the ongoing, complex debate about the ethical limits of brain organoid research.^14 15^

## Biological revolution

This research exists against a backdrop of radical genetic modification of animals for human purposes which has been going for centuries, beginning with the breeding of dogs from a small group of canids and wolves. For decades, animals have been genetically modified to suffer from human diseases to enable medical experimentation. Mouse models have provided insight into human ‘cancer, heart disease, hypertension, metabolic and hormonal disorders, diabetes, obesity, osteoporosis, glaucoma, skin pigmentation diseases, blindness, deafness, neurodegenerative disorders (such as Huntington’s or Alzheimer’s disease), psychiatric disturbances’ and more.^16^

Indeed, recently a genetically modified pig heart was transplanted into a human, David Bennett, but he died after 2 months—possibly due to a virus from the pig.^17^ Human-pig chimaeras have also been created using stem cell technology and genetic engineering (‘blastocyst complementation’) to create human organs (livers, pancreas, etc) in pigs with the hope of transplanting these organs to humans.^18^ These chimaeras could have biologically humanised body parts, including brains made of some human neurons, and some pig neurons, in some ways similar to *The Fly*.

As this technology progresses, it will be tempting to move this research to primates, who are closer to humans than rats. This would give a more accurate picture of brain development. However, because primates are so much closer to us in biological terms, it may be easier to be fully ‘morally humanised’. (Arguably, higher primates like Great Apes should already be accorded full moral status as they are self-conscious, rational and have moral capacities.^19^

A number of factors would determine how likely this is: the size of the brain, how early in development of the recipient the organoid is transplanted, the size of the organoid and the areas of the recipient brain which it affects.^20^ One group has proposed a study that attempts to cross the human/animal species barrier and to create humanised brains in monkey brains using the method of blastocyst complementation. However, they argue that a step-by-step research procedure should be followed to avoid the monkey’s cognitive functions being enhanced as a result of the study.^21^

We are in the midst of a biological revolution harnessing the engine of biology to serve our needs. We are even creating life synthetically.^22^

## The answer: function, not structure

Many people will be tempted to discount the moral status of these new life forms. After all, are not these organoids just derived from skin cells? Also, are not animals implanted with these organoids no different from conventional chimeric animals containing human-derived materials?

The origin of a neuron, brain or person does not matter morally. It does not matter whether they are natural or artificial, carbon based or silicon based. Their anatomy or structure does not matter. Their appearance does not matter.

What matters morally is function. The rats receiving human brain organoids did not have enhanced cognitive functions. As the *New York Times* reported,

Giorgia Quadrato, a neurobiologist at the University of Southern California who was not involved in the new study, noted that the human organoids did not make the rats more human. On learning tests, for example, they scored no better than other rats.

‘“They are rats, and they stay rats,’ Dr. Quadrato said. ‘This should be reassuring from an ethical perspective’.^23^

However, as this research progresses, it is essential to assess function.^24^ What kinds of function?

First, consciousness is sufficient condition for attributing some moral status. In this context, it doesn’t matter how you treat a rock. It matters how you treat a rat. As early as 1789, Bentham posed the question, ‘The question is not, Can they reason?, nor Can they talk? but, Can they suffer? Why should the law refuse its protection to any sensitive being?’^25^

If a being is conscious, it should not have pain inflicted on it without good reason. This is the reason for the Animal Liberation movement^26^—they believe the pleasure of eating animals is not a good reason for the suffering incurred in factory farming. In the current experiment, Pasca claimed the rats did not show any evidence of suffering.^23^

Second, it is wrong to kill an animal if it has features definitive of human beings, as this would suggest that the animal bears full moral status. There is division over what this property is: self-consciousness, rationality, moral capacity, advanced empathy.^24^ These properties are seen as sufficient conditions for attributing full moral status to a being.

But the key point is that if function, not structure, matters, it is necessary to test the behaviour and mental functions of new life forms before they are sacrificed for human purposes. This requires functional experiments to test morally relevant functions in tandem with these ground-breaking experiments to study disease.^15 27^ As a result, mere consciousness might warrant some moral status, thereby demanding the same ethical framework as standard animal experiments. This perhaps aligns with Pasca’s assertion that the animals resulting from his experiments did not exhibit human-like suffering. However, should an entity possess human-like attributes, it would necessitate moral considerations on par with those granted to humans.

## Reproductive cloning issues resurface?

Brain organoids, derived from iPS cells, can be a clone of an existing human person insofar as they are genetically identical to the cell donor.^28^ They take a person’s genetic programme from a skin cell and build a brain from that. If brain organoid research were to progress and substantial parts of key areas of, say, primate brains were replaced. If this replacement results in self-consciousness at the site of transplantation, or if the consciousness of the transplanted human brain organoid coexists with that of the host primate brain, we may confront a daunting ethical dilemma: the existence of human consciousness within a primate’s body.

The brain determines what matters most in our lives—our desires, hopes, dreams, expectation, love, rational capacities, ability to enjoy relationships with others, and so on. A clone of a person’s brain could be a clone of that person, if that brain were able to function. Now this is not a clone or copy of a whole brain. If the technology progresses to build a more intricate or a full brain model, it could rekindle the controversies surrounding reproductive cloning—a topic that garnered substantial global debate over two decades ago and led to widespread regulation.

## Law, regulation and moral status

It is technically possible to imagine an organoid with a complete brain structure and various conscious experiences. Let’s call Animus a full brain, a fully conscious organoid. What is at one point science fiction, quickly becomes a reality, as the gene editing of Lulu and Nana shows.

It is likely that most countries would not approve research to create a human brain in a non-human animal, no matter what scientific or medical purpose it may serve.^29^,^31^ But is it illegal?

Some countries have formed stringent laws on creation of clones, heritable gene editing and chimaeras. But these ingenious experiments may take a different path than those anticipated in these laws, which rapidly become outdated.^15 32^ Previous laws anticipated ethical issues around tissue as it related to the tissue’s owner, not to the interests of the tissue itself. Other laws related to embryonic stem cells, but not those generated from skin cells. It is essential that ethics keeps pace with science. Science and ethics must work together.

One response is to create more nimble legal instruments. This might involve international regulatory advice bodies that involve expert scientists and ethicists. The WHO would be one venue though the kinds of products they produce are typically cumbersome, conservative and not forward looking. Moreover, they lack deep ethical expertise, focusing more on geographical representation.

One kind of force which could have international penetration is peer or social pressure. Scientific bodies and journals could refuse to publish or acknowledge unethical research.

One example is the International Society for Stem Cell Research (ISSCR) including leading scientists in the field. This body has been a leader in the field, including the service of professional ethicists. It has been proactive, providing regularly updated guidelines which include consideration of ethical issues.

The ISSCR produced updated guidelines in 2021. For an analysis, see Koplin.^33^ Koplin argues that these guidelines essentially put chimaeras under existing animal research regulations and these are inappropriate.

Importantly these guidelines are already out of date. They eschew the possibility of organoids being conscious… But these chimaeras are clearly conscious so at very least organoids are contributing to a conscious brain.

Koplin’s argument against the ISSCR guidelines centres on the fact that possessing consciousness and self-consciousness are necessary conditions for some and full moral status, respectively. In contrast, the ISSCR posits that the presence of consciousness is a sufficient condition for some moral status and dismisses the near-term potential for organoids or animals implanted with human brain organoids to attain consciousness or cognitive enhancement.^6 34^

These are topics of great philosophical complexity that warrant book length treatments. The point for our purposes is that we are still in the ethical wilderness. The biggest ethical issues involved in the biological revolution relate to the nature of well-being, moral status^35^, identity, the value of existence and what we owe each other.^36^ These issues have not been resolved in relation to applies even to non-human animals,^24^ let alone these novel life forms.

In response to these challenges, it is imperative that we allocate as much time and human resources to the ethics as we do to the science. With the advent of formidable technological power, it is incumbent on us to discern its ethical and appropriate utilisation.

## Data Availability

There are no data in this work.
